# Capsaicinoids Modulating Cardiometabolic Syndrome Risk Factors: Current Perspectives

**DOI:** 10.1155/2016/4986937

**Published:** 2016-05-23

**Authors:** Vijaya Juturu

**Affiliations:** OmniActive Health Technologies Inc., 67 East Park Place, Morristown, NJ 07960, USA

## Abstract

Capsaicinoids are bioactive nutrients present within red hot peppers reported to cut* ad libitum* food intake, to increase energy expenditure (thermogenesis) and lipolysis, and to result in weight loss over time. In addition it has shown more benefits such as improvement in reducing oxidative stress and inflammation, improving vascular health, improving endothelial function, lowering blood pressure, reducing endothelial cytokines, cholesterol lowering effects, reducing blood glucose, improving insulin sensitivity, and reducing inflammatory risk factors. All these beneficial effects together help to modulate cardiometabolic syndrome risk factors. The early identification of cardiometabolic risk factors can help try to prevent obesity, hypertension, diabetes, and cardiovascular disease.

## 1. Introduction

Obesity is becoming an epidemic condition and tends to raise diabetes, coronary heart disease, hypertension, and inflammatory disorders. Increase in obesity rates increases healthcare costs to billions of dollars for different obesity associated chronic conditions. Diabetes is a major risk factor in 347 million people in worldwide population and it may increase by 2030 and will be the leading cause of death [1]. Hypertension is another major risk factor and independently associated with hyperglycemia, renal failures, and nephropathy in diabetes, endothelial dysfunction, and all-cause mortality. CHD risk increases by 29% and stroke risk increases by 46% in people with an increase in diastolic blood pressure of 7.5 mmHg. Individuals who steadily lose about 1 to 2 lbs. per week are more successful at keeping weight off and every pound of weight loss may reduce the risk of diabetes by 16% [2]. There are few modifiable and nonmodifiable risk factors associated with risk factors of obesity to prevent overweight and obesity. Herbs and spices in the diet are known for potential health benefits. Identifying alkaloids, efficacy of biologically active substances in herbs and spices, or their physiological properties, including efficacy, doses, and safety issues are important to identify its role in modulating risk factors. Herbs and spices can be used in meal cuisines and recipes to partially substitute other ingredients such as table salt, table sugar, added disaccharides (fructose, sucrose), and saturated fatty acids and may create more appetizing dishes to increase flavor and taste. In a prospective cohort study, men (*N* = 199,293) and women (*N* = 288,082) in China aged 30 to 79 years were examined for the relationship between the regular consumption of spicy foods in their meals and deaths due to specific and associated chronic conditions such as diabetes, CHD, and respiratory disorders [3]. Spicy food consumption showed highly significant inverse relationship with deaths (14% relative risk reduction) among both genders based on potential risk factors due to cancer, CHD, and respiratory disorders. Recent studies in animals and humans have shown that herbs and spices have potential beneficial effects such as anti-inflammatory, cholekinetic, antioxidant potentials, antiatherosclerotic effects, anticarcinogenesis, antitumorigenesis, no genotoxicity, and no mutagenesis, protect liver and kidneys, and reduce micro- and macrovascular complications of diabetes and dyslipidemia [4].

**Figure 1:**
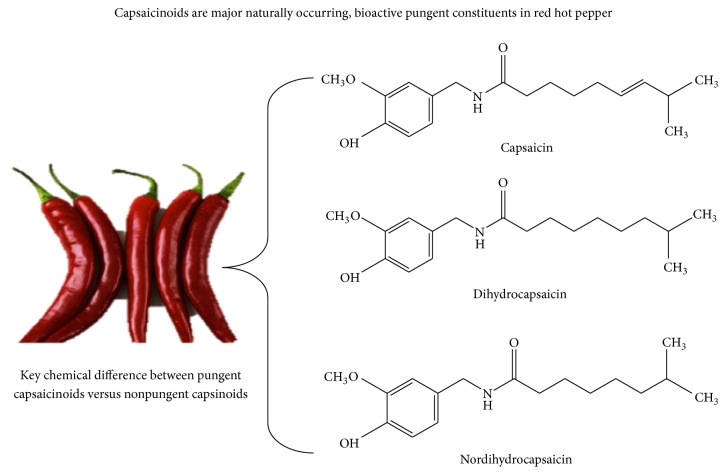
The structure of the capsaicinoids.

Evidence shows that abdominal adiposity may decrease and fatty acid oxidation increases with red and long-term consumption of spicy foods decreasing the incidence of CMS [5, 6]. Capsaicin/capsaicinoids (CAPs) are found to exert physiological properties and efficacy including the activities of the loss of the ability to feel pain, thermogenesis, lipolysis, no genotoxicity, no carcinogenesis, reducing oxidative stress, anti-inflammatory properties, and antioxidant properties. Natural source of CAPs is mainly from chili, cayenne pepper, and red pepper and regular human consumption was reported to be 2.5 g–20 g/day [7, 8]. This review summarizes the effects of CAPs and its modulating effects on cardiometabolic syndrome risk factors. Capsaicin (80%) and dihydrocapsaicin (90%) are the major components of capsaicinoids (CAPs) (Figure 1) and ratio is around 1 : 1 and 2 : 1 [9]. Cardiometabolic risk has been defined as a cluster of risk factors for cardiovascular disease (CVD) characterized by glucose intolerance or diabetes, abdominal obesity, hypertension, and dyslipidemia. The metabolic, homodynamic, and renal abnormalities contribute to cardiometabolic syndrome (CMS).

There are many varieties of spices chili pepper or “chilies,” sweet bell pepper, banana pepper, trinidad perfume chili pepper, cubanelle pimiento, cherry pepper, Tuscan pepper, poblano, coronado pepper, NuMex R Naky chili, panca chili, and so forth. In North America, red pepper, green pepper, or bell pepper are commonly used, while in New Zealand, Australia, and India it is typically called “capsicum.” Capsicum has been safely consumed in large amounts in many countries, especially in Mexico and Korea, where per capita consumption can reach 15 grams per day. More moderate consumption of capsicum [up to 5 grams daily per capita] is seen in countries like Thailand and India. In contrast, North Americans and Europeans consume on average less than 1 gram per day. Some people enjoy the heat and the demand for CAPs spiced food and beverages has a long and colorful history [10]. CAPs may have potential biological and therapeutic properties in the management of gaining weight and weight management, cardioprotective influence, antilithogenic effect, and anti-inflammatory, analgesic, thermogenic, and digestive stimulant action and modulation of intestinal ultra-structure so as to enhance permeability to micronutrients in the gastrointestinal (GI) system. It has been shown that CAPs are potential agonists of CAPs receptor (TRPV1).

The substances that give hot chili peppers their intensity or pungency when ingested or applied topically are collectively called CAPs [11]. In 1912, Scoville organoleptic scale was developed by an American pharmacist Wilbur Scoville. The Scoville organoleptic scale is a measure of the perceived heat intensity from a chili pepper (Table 1). The Scoville scale is a taste detection based method for rating the heat of chili peppers. A measured amount of pepper extract has sugar added to it incrementally until the heat is undetectable through taste. Though it is on imprecise method, it has been estimated that 1 unit corresponds to 18 *μ*M.

These are hydrophobic, colorless, odorless, and crystalline to waxy compounds, a variety of which are present in capsicum. The most abundant CAPs (Figure 1) are CAPs [8-methyl-N-vanillyl-6-nonenamide], dihydrocapsaicin/capsaicinoids, and nordihydro CAPs [12]. Approximately 1 g of dried red pepper provides 3 mg of CAPs [13]. Recent reviews and Met analysis suggest that calorie intake was reduced with a minimum dose of 2 mg of CAPs per day and reduced energy intake by 74 kcal (310 kJ)/meal (*P* < 0.001) and helps in weight management [14, 15].

## 2. Overweight/Obesity

Obesity, due to its increasing incidence around the world, has been considered an epidemic in developed and developing countries. Energy imbalance, environmental, sedentary life, sociocultural changes, and quality of life are the fundamental causes of obesity and overweight. Normal body fat percentage of a healthy man was 15–20%, while normal body fat for healthy women was approximately 25–30%. If a person gains 20% over their body mass index (BMI), he/she is considered as obese. Body fat (BF) percentage was calculated based on BMI, age, and gender using Deurenberg equation, as follows:

(1) body  fat  percentage=1.2BMI+0.23age−10.8sex−5.4.

Age and sex are designated as 1 for males and 0 for females. BMI is typically associated with body fat percentage. So it is necessary to calculate BMI and BF for weight management. Fat distribution based on android obesity is associated with central obesity with excess fat in the abdominal wall and visceral mesentery is strongly associated with cardiometabolic consequences. CAPs are also known to stimulate energy expenditure to increase the rate of sympathetic nervous system activity which induces catecholamine [e.g., adrenaline] secretion from the adrenal medulla [16–19]. This thermogenic effect has been exploited for purposes of weight management. CAPs have been reported to reduce appetite [20–22], to increase thermogenesis [13, 23–25], and to increase lipolysis [13, 15, 23, 26, 27], or decrease WHR and appetite [28] by serum glycerol and free fatty acids [29]. CAPs increased satiety and reduced energy and fat intake. Satiety (area under the curve) significantly (*P* < 0.01) increased from 689 to 757 mmh in the men and from 712 to 806 mmh in the women and the AUC for hunger decreased after ingestion of CAPs. Average daily energy intakes were 10% lower after the red pepper in capsules [0.9 g red pepper (0.25% capsaicin; 80,000 Scoville Thermal Units)] and 16% lower after the red pepper in tomato juice compared with the placebo groups [22]. The science supports an association between capsaicin/capsaicinoids (CAPs), containing food consumption and a lower incidence of obesity [6, 16].

### 2.1. Metabolism

Metabolism is categorized as catabolism and anabolism. Energy formation is one of the vital components of metabolism. The metabolic pathways rely upon nutrients that they breakdown in order to produce energy. CAPs are absorbed through the GI tract by a passive transport across membranes and then circulated systemically via plasma [30]. CAPs are believed to stimulate thermogenesis by activating the sarcoplasmic reticulum Ca^++^ATPase [SERCA]. Thermogenesis refers specifically to heat generation via SERCA ATP hydrolysis. In the presence of CAPs SERCA becomes uncoupled [ATP hydrolysis occurs without calcium transport] leading to the generation of heat [31]. The thermogenic effect of CAPs is mediated, at least in part, by a CAPs sensitive structure located in the rostral ventrolateral medulla [32]. CAPs treatment may also stimulate vasodilation [33].

Consumption of red pepper has been shown to promote negative energy balance [34]. The dose of red pepper consumption is up to 10 g in south Asian countries and exceeds the amount consumed by the general US population ~1 g in their meals. In another randomized crossover clinical trial, 25 young healthy adults consumed 0.3, 1, or 1.8 g of red pepper per meal. An increase in postprandial energy expenditure (EE) and core body temperature and a decrease in skin temperature were observed with 1 g versus 0.3 g intake of red pepper. Respiratory quotient [VCO_2_/VO_2_] was lower after the 1.8 g dose was ingested orally versus in capsule form. TRPV1 activation by CAPs improves energy metabolism through PGC-1*α* [35]. Thus, these results suggest the effects of red pepper on metabolic and sensory inputs. It was also suggested that individuals may become desensitized (maybe a plateau level) to red pepper with long-term consumption of spicy foods [36].

Regarding appetite suppression and analgesia, CAPs bind to a receptor called the vanilloid receptor subtype 1 [VR1] [37]. TRPV1, a heat-activated calcium channel was stimulated with either heat or physical abrasion and thus allows cations to pass through the cell membranes. CAPs in extreme exposure may cause a chemical burn [38, 39].

The effects of CAPs in adipose tissue and liver are associated with PPAR-alpha and TRPV-1 expression/activation [40]. Several proteins are altered by CAPs, many of which suggest increased metabolism. CAPs treated with high-fat diet in rats reported 8% decrease in body weight over controls. There was downregulation of heat shock protein 27 [Hsp27] and Steap3 protein and upregulation of olfactory receptor [Olr1434] in which levels of vimentin, peroxiredoxin, and NAD[P]H: Quinone oxidoreductase 1 [NQO1] were significantly reduced >2-fold, whereas aldo-keto reductase and flavoprotein increased with CAPs. Joo et al. [41] data demonstrate that CAPs alter thermogenesis and lipid metabolism related proteins and it may be a useful phytochemical for weight management.

CAPs absorbed from the gut lumen are almost completely metabolized before reaching the general circulation yet regulate adipose tissue distribution. A CAPs sensitive intestinal mucosa afferent mechanism seems to modulate body fat distribution [42]. In vitro, CAPs decreased the intracellular lipid content and increased glycerol release in a concentration-dependent manner in adipocyte cell culture. Leung [42] reported that hormone sensitive lipase [HSL], carnitine palmitoyl transferase-I*α* [CPTI-*α*], and uncoupling protein 2 [UCP2] genes were upregulated significantly and these genes are involved in lipid catabolism. These results suggest that CAPs affect lipolysis through lipid catabolism, including thermogenesis [i.e., UCP2] [41]. Meta-analysis findings [14, 15] showed that CAPs administration reduced calorie intake by 309.9 kJ (74.0 kcal, *p* < 0.001). It was observed that a minimum dose of 2 mg of CAPs are needed to reduce calorie intake. Absorption of CAPs was about 94% and maximum distribution of 24.4% of CAPS was observed at 1 hour. Only a small amount of absorbed CAPs [<0.1%] was excreted in urine [43]. The tested thermogenic potentials of products in humans range from marginal to modest, that is, 2–5% above daily energy expenditure. Some of these ingredients could be increased to 10–15% above daily energy expenditure and may have a clinically significant impact on weight management for postslimming weight maintenance to avoid weight recycling [44]. Overall, thermogenesis due to CAPs may be due to *β*-adrenergic stimulation [29], reduction in calorie intake, and enhanced energy expenditure and lipid (Table 2).

### 2.2. Free Fatty Acid and Lipolysis

Triglycerides are hydrolyzed into glycerol and free fatty acids released from the fat cells during lipolysis. The lipolysis pathway and FFA reesterification may lead to a cycle for energy turnover. Ingestion of 2 mg capsaicinoids (CAPs) from Capsimax® was associated with an increase in blood FFA and glycerol at selected postingestion time points compared to placebo [29]. TRPV1 activation by CAPs reduces vascular lipid accumulation [45]; prevents nonalcoholic fatty liver [46]; and improves visceral fat remodeling [47]. CAPs associated with an increase in circulating free fatty acids due to beta-adrenergic stimulation.

Hormone sensitive lipase (HSL) is the major lipase for lipolysis. It is an essential enzyme capable of hydrolyzing triglycerides, lipids, and cholesteryl esters and abnormal expression of this gene leads to CMS. Obesity may be due to metabolic disturbances in pathways of thermogenesis, lipolysis, and lipid metabolism. In addition, downregulation of catecholamine-mediated lipolytic response may increase adiposity in tissues. Increase in lipolysis may enhance circulating NEFA concentrations in blood and may enhance CMS complications. Epinephrine (EPI) and norepinephrine (NE) interact with hormone sensitive lipase; an increase in triglyceride degradation may be observed with capsaicinoid intake, which may lead to an increase in circulating free fatty acids (FFA) and glycerol. FFA and glycerol concentrations were relatively higher in dihydrocapsaicin (DHC) than in capsaicin (CAP). In 2014, Ludy and Mattes [36] reported test loads with a 1 g red pepper dose enhanced thermogenesis and decrease in orexigenic sensations, compared with no hot red peppers (RP) and a decrease in respiratory quotient (RQ). Further, a preferred RP dose (range 0.1–3.5 g) was associated with a lower respiratory quotient (RQ) indicating that an increase in fat oxidation was observed with a RP dose range 0.1–3.5 g. These results suggest potential thermogenic effects of RP.

### 2.3. Appetite

Body weight is regulated by complex homeostatic mechanisms. Even a subtle mismatch (less than 0.5%) in caloric intake over expenditure is sufficient to cause weight gain [44]. A decrease in appetite and intake of protein and fat intakes were observed with red pepper consumption in females and calorie intake in males [20]. Dietary supplementation of 2 mg CAPs for 12 weeks decreased WHR and appetite in healthy individuals [28]. CAPs in the diets increase satiety and fullness and reduce food intake [48]. After dinner, capsaicin prevents the effects of the negative energy balance on desire to eat. In a recent open label study [49], one hundred fifty active individual healthy subjects were studied for a week with supplementation with 2 mg CAPs from capsicum extract (Capsimax) reported 7% statistical significant decrease in appetite. Further studies are warranted in double blind placebo controlled studies. Thus CAPs are a natural source that effectively inhibits adipogenesis and activates adipocyte cycle at various levels and potentially stimulates lipolysis activity.

### 2.4. Respiratory Quotient (RQ)

Respiratory quotient (RQ) measures the ratio of carbon dioxide (CO_2_) production to oxygen (O_2_) consumption, under steady fasting and resting conditions. RQ is useful measure because the volumes of CO_2_ and O_2_ produced as fuel source are being metabolized under different environmental conditions. Janssens et al. [50] demonstrated that respiratory quotient (RQ) was more decreased at 75% capsaicin (CAPS, *P* = 0.04). These results suggest that an enhancement of energy metabolism may be due to a direct (as an agonist) and/or an indirect (via catecholamine) beta-adrenergic action. Further studies are required to explore RQ action of capsaicin.

### 2.5. Carbohydrate Oxidation

Indirect calorimetry (hood system) is used to measure the total carbohydrate oxidation whereas exogenous carbohydrate oxidation is estimated from carbon dioxide production (VCO_2_). Lim et al. [51] demonstrated enhanced RQ and blood lactate levels in the presence and absence of physical activity with hot red pepper in a meal. At 30 minutes after the meal consumption, oxygen consumption was nonsignificantly higher at rest. Significantly higher plasma epinephrine and norepinephrine levels were observed at 30 min after the meal. These results suggest that hot red pepper ingestion stimulates carbohydrate oxidation. In another study, Yoshioka et al. [23] reported that carbohydrate oxidation was significantly decreased by the addition of red pepper in a meal. It was concluded that CAPs could favorably modulate metabolism and possess beneficial effects on thermogenesis, lipolysis, insulin resistance, satiety, resting energy expenditure (REE), respiratory quotient, glucagon-like peptide-1 (GLP-1), free fatty acids (FFA), and glycerol release and regulate adipose tissue distribution.

## 3. Diabetes

Improving insulin sensitivity by increasing the rate of fat oxidation independently reduced food intake. Current data suggest that nearly 50% of adults living in the US have diabetes or prediabetes. Diabetes can damage micro- and macrovascular circulation (blood vessels, nerves, the eyes, and kidneys), diabetic foot, poor wound healing, and devastating soft tissue infections. Recent studies have shown a metabolic role of CAPs that may be mediated via the transient receptor potential vanilloid type-1 (TRPV1) channel [52]. The stimulation of adrenergic, thyroid hormone, or growth hormone receptors; the inhibition of glucocorticoid receptors; the modulation of transcription factors [e.g., peroxisome proliferator-activated receptor delta (PPAR delta, PPAR-alpha activators) activators] or enzymes [e.g., glutamine fructose-6-phosphate amidotransferase (GFAT) inhibitors, AMP-activated protein kinase], promoting mitochondrial biogenesis and fatty acid oxidation, are some of the approaches of thermogenesis. Capsicum has been shown to help improve metabolism and hormone function [39] and stabilize blood glucose; successful body mass loss was associated with higher initial body mass [26] and reduced insulin and leptin resistance [39]. TRPV1 activation by CAPs improves glucose homeostasis [40]. It was concluded that CAPs could favorably modulate diabetes risk factors and possess beneficial for glucose management. Further research is to test the dose effect on metabolic health risk factors and tolerability in health and disease condition. It is also necessary to study the interaction with medications and/or an adjuvant therapy.

## 4. Hypertension

Blood pressure is a measurement of the force applied to the walls of the arteries as the heart pumps blood through the body (heart muscle contracts versus heart muscle relaxes). The normal or abnormal blood pressure is determined by the force and amount of blood pumped and the size and flexibility of the arteries. Hypertension is independently associated with CHD, stroke, renal disease, and all-cause mortality. CHD risk increases by 29% and stroke risk increases by 46% in people with an increase in diastolic blood pressure of 7.5 mmHg. In the United States, 1 out of 3 reported high blood pressure and it is now epidemic across the country. Spices have shown its effects in reducing renovascular hypertension. Liang et al. [53] reported that CAPs could improve endothelial function by inhibiting the gene expression of COX-2. NO inhibits nearly all enzyme-catalyzed reactions such as protein oxidation, increases blood flow to insulin-sensitive tissues, and increases the phosphorylation of HSL and perilipins, leading to lipolysis. The properties of NO are expression of peroxisome proliferator-activated receptor-gamma coactivator-1alpha, enhancing mitochondrial biogenesis and oxidative phosphorylation. Yang et al. [54] reported CAPs could activate PKA and eNOS in the endothelia by activation of TRPV1 in spontaneous hypertensive rat (SHR) model. Potential mechanisms of CAPs may be due to vascular tone integrity and other factors may be due to the release of calcitonin gene-related peptide (CGRP), substance P (SP), and neurokinin A [55–57], an increase in intracellular calcium, promoting nitric oxide release and lowering blood pressure [58], and release of neuropeptides [58], such as substance P and calcitonin gene-related peptide (CGRP) [57, 58]. Other mechanisms may be through brown adipose tissue (BAT) including oxidation of fatty acids and glucose [59]. TRPV1 activation by CAPs lowering blood pressure through promotes urinary sodium excretion [60] and delays the onset of stroke [61]. TRP channels may disturb intracellular calcium ([Ca(2+)]i) homeostasis and lead to endothelial dysfunction for the development of high blood pressure/hypertension [62, 63].

Overall, CAPs may stimulate expression of peroxisome proliferator-activated receptor-*γ* coactivator 1 (the master regulator of mitochondrial biogenesis), nitric oxide synthase, heme oxygenase, and adenosine monophosphate-activated protein kinase. CAPs may increase endothelial function, blood flow to tissues, lipolysis, and the catabolism of glucose and fatty acids, inhibit fatty acid synthesis, and reduce oxidative stress, thereby improving overall metabolic health profile.

## 5. Coronary Heart Disease

Coronary heart disease (CHD) mortality rates dropped in the past four decades but prehypertension and prediabetes prevalence increased in children and adults. Rate of obesity, diabetes, and hypertension with change in quality of life including sedentary life, use of electronic devices, and stress increased risk in all age groups. CAPs role on plasma lipid profile and cholesterol levels, aortic function including atherosclerotic plaque development, cholesterol absorption, excretion of fecal sterols, and up- and downregulation of gene expression of major receptors, enzymes, and transporters involved in cholesterol and fatty acid metabolism was reported in animals [64–66]. Huang et al. [65] demonstrated that TRPV1 gene deletion may result in inflammation and cardiovascular dysfunction due to disproportional left ventricular remodeling. Hence TRPV1 may prevent myocardial infarction and injury by inhibiting inflammation pathways and abnormal tissue remodeling in rats. TRPV1 activation by CAPs attenuates high-salt diet-induced cardiac hypertrophy [67, 68].

In humans, CAPs have also been linked to cardiovascular health, by improving endothelial function [33], activating brown fat thermogenesis and reducing body fat [64], and inhibiting LDL-cholesterol oxidation [66], and increase the resistance of serum lipoproteins to oxidation [69]. CAPs reduce atherosclerosis and cholesterol absorption, maintain aortic integrity, reduce lipids in blood, decrease ratio of plasma campesterol/cholesterol, and increase fecal excretion of sterols in animal models [53, 65]. In another study [70], CAPs downregulated the mRNA levels of hepatic 3-hydroxyl-3-methylglutaryl CoA reductase and cholesterol-7*α*-hydroxylase and upregulated transient receptor potential vanilloid type-1, ileal apical sodium-dependent bile acid transporter, and intestinal bile acid binding protein. Inhibition of hepatic cholesterol synthesis was due to the decrease in the excretion of small intestinal bile acid contents and fecal bile acid [71]. CAPs displayed a strong potent inhibitory effect of pancreatic lipase (PL) compared to Orlistat. IC50 for CAPs from Capsimax was 5.4 (*μ*g/mL) compared to Orlistat (0.53 *μ*g/mL). Capsimax enhanced lipolysis after 24 h treatment. The ratio of released glycerol/protein content was 1.59, compared to positive control, isoproterenol (ratio: 1.60). Capsimax reduced fat accumulation by downregulating PPAR*γ* and C/EBP*α* in 3T3Li adipocytes [72]. The hypocholesterolemic activity of CAPs may be due to conversion of cholesterol to bile acids and an increase in fecal total bile acid excretion. It was concluded that CAPs could favorably modulate plasma lipids and possess beneficial vascular activity and cholesterol management.

### 5.1. Endothelial Function/Vascular Tone

The endothelium is the thin layer of cells that line the interior surface of blood vessels, forming an interface between circulating blood in the lumen and the rest of the vessel wall, and maintains vascular homeostasis. Endothelium-derived relaxing factors maintain vascular tone and integrity in the presence of NO. TRPV1 channels are observed in the heart and especially at myocardium and the circulatory system, smooth muscle cells, and endothelial cell lines [73, 74]. CAPs-induced vasodilatation decrease TRPV1 and cation influx expression [75]. Vascular tone regulation is maintained by the activation of TRPV1, phosphorylation of protein kinase A (PKA), and eNOS. It was observed that CAPs may contribute to the physiological regulation of vascular tone. Further studies are required to study the dose effect on vascular tone and for the vascular integrity.

### 5.2. Inflammation and Oxidative Stress

Obesity is an epidemic chronic disease of multifactorial origin. Factors associated with current obesity prevalence are due to sociocultural, behavioral, psychological, metabolic, cellular, and molecular factors. Inflammation is a primary risk factor due to oxidative stress, which is also implicated in the progression of obesity, diabetes, hypertension, and atherosclerosis conditions. Chronic exposure to CAPs reduces pain sensation and blockade of inflammation due to its association with neurotransmitters [76, 77]. A projection of the National Cancer Institute (NCI) that medical health care cost for obesity and cancer will be increased in 2030 and it will add half a million cases of cancer in the United States [78]. Capsicum may also help prevent cancer, likely due to its antioxidant activity [79–81]. CAPs-induced TRPV1 activation could enhance the PKA-UCP2 pathway, thus improving mitochondrial function, reducing ROS production, and ameliorating atherosclerosis [82].

Overall, CAPs could favorably modulate plasma lipids and metabolic health risk factors and maintain vascular tone and vascular integrity, decreasing the pad weights of epididymal and prerenal adipose tissues. Current studies suggest that CAPs may reduce CHD risk factors, improve endothelial function, maintain aortic functionality, reduce atherosclerotic plaque development, and inhibit fat and cholesterol absorption and gene expression of major transcription receptors, enzymes, and transporters involved in cholesterol metabolism in animal models.  Table 3 provides a summary of CAPs role in modulating cardiometabolic risk factors. Counseling on lifestyle interventions, including reducing fast food intakes, heavy alcohol, smoking, increase in physical activity, a heart-healthy no added sugar or salt diet, and weight maintenance, is important for all people regardless of risk level, even if it is only to reinforce established healthy quality of life. A reduction in 1 percent of body mass index (BMI) is equivalent to a weight loss of roughly 1 kg (or 2.2 lbs) for an adult of average weight along with exercise reported to reduce risk of cancers [78] and 5–10% weight loss will reduce risk of cardiometabolic syndrome [83]. Further studies are required to explore CAPs role in human cholesterol metabolism and CMS risk factors. Safety, effectiveness, or interactions with medications (prescription or nonprescription) are very important for CMS risk factors.

## 6. Safety

Carcinogenic and genotoxicity studies and animals and human clinical trials to date have reported no adverse effects with CAPs. CAPs are well-tolerated in controlled human clinical trials, as ingredient alone or in combination with other ingredients with and without meal at low to high doses in short-term and long-term studies. However, some people are not tolerant with CAPs with some gastrointestinal symptoms; this phenomenon is quite common [84]. Long-term randomized clinical trials on CAPs are needed to explore its role in glucose and lipid metabolism in human health and disease conditions.

## Figures and Tables

**Table 1:** Scoville heat units of different chili pepper.

Scoville heat units	Chili pepper
16,000,000	Pure capsaicin and dihydrocapsaicin
9,100,000	Nordihydrocapsaicin
8,600,000	Homodihydrocapsaicin and homocapsaicin
2,000,000	Common pepper spray
1,001,304	Naga-Bih Jolokia pepper
100,000–350,000	Habanero (*Capsicum chinense *Jacquin)
100,000–325,000	Scotch bonnet (*Capsicum chinense*)
100,000–225,000	Birds eye pepper
100,000–200,000	Jamaican hot pepper
100,000–125,000	Carolina Cayenne pepper
95,000–110,000	Bahamian pepper
85,000–115,000	Tabiche pepper
75,000–80,000	Red amazon pepper
75,000	Chile-Today Red Amazon Powder, from Chile-Today Hot Tamale
50,000–100,000	Thai pepper (*Capsicum annuum*)
50,000–100,000	Chiltepin pepper
40,000–58,000	Piquin pepper
40,000–50,000	Super chili pepper
40,000–50,000	Santaka pepper
30,000–50,000	Cayenne pepper (*Capsicum baccatum* and *Capsicum frutescens*)
30,000–50,000	Tabasco pepper (*Capsicum frutescens*)
15,000–30,000	De Arbol pepper
12,000–30,000	Manzano pepper
6,000–23,000	Serrano pepper
5,000–10,000	Hot wax pepper
2,500–8,000	Santaka pepper
2,500–5,000	Jalapeño (*Capsicum annuum*)
2,500–5,000	Guajillo pepper
1,500–2,500	Rocotillo pepper
1,000–2,000	Pasilla pepper
1,000–2,000	Ancho pepper
1,000–2,000	Poblano pepper
700–1,000	Coronado pepper
500–2,500	Anaheim pepper
500–1,000	New Mexico pepper
400–700	Santa Fe Grande pepper
100–1000	Cubanelle pepper (*Capsicum annuum*)
100–500	Pepperoncini, pepper also known as Tuscan peppers, sweet Italian peppers, and golden Greek peppers
100–500	Pimento
0	Sweet bell pepper

Source: http://www.chilliworld.com/FactFile/Scoville_Scale.asp.

**Table 2:** Capsaicinoids effects on potential markers of weight management.

Markers (number of positive studies/total studies reported)	Capsaicin/capsaicinoids dose^*∗*^	Potential biological effects
Energy expenditure (*N* = 18/20 studies)	3–30 mg/d	Increase in EE for 30 min [12, 22–25, 27, 36, 51, 64], [15]^*∗*^

Lipid oxidation and fat loss (15/18 studies)	1–30 mg/d	Increase in lipid oxidation and decreased abdominal/body fat [13, 18, 22–24, 27], [15]^*∗*^

Lipolysis (1/1)	2 mg	Tolerable dose, increased plasma FFA (2 hr and 2.5 hr), and glycerol (4 hr) concentrations [16]

Appetite (7/9 studies)	2–30 mg	Decrease in appetite, decrease in energy intake, and increase in satiety [13, 20–23, 28, 36, 49]

Weight management markers (3/3)	2 mg	Body indices (waist circumference, hip circumference, and WHR); appetite; lipolysis [16, 28, 49]

^*∗*^Cross reference.

**Table 3:** Summary: capsaicinoids modulating CMS risk factors through different potential mechanism of action.

CMS markers	Potential mechanism of action^*∗*^
Control of IGT/diabetes	Reducing carbohydrate absorption, improving insulin sensitivity, improving glucose utilization and metabolism, delaying glucose absorption, increase in fecal excretion of acidic sterols, decreasing fasting and postprandial hyperglycemia and improving glycemic control, increasing blood flow to insulin-sensitive tissues, adipose tissue lipolysis, and the catabolism of glucose and fatty acids but inhibiting fatty acid synthesis, and ameliorating oxidative stress [26, 39, 40]

Reduction of dyslipidemia	Antilipidemic effects: binding bile acids and inhibiting pancreatic lipase; plasma lipids, functionality of aorta including atherosclerotic plaque development, cholesterol absorption biomarker, mRNA levels of hepatic 3-hydroxyl-3-methylglutaryl CoA reductase, and cholesterol-7*α*-hydroxylase were downregulated; upregulation of cholesterol 7*α*-hydroxylase and downregulation of liver X receptor alpha, inhibiting LDL-cholesterol oxidation, fecal sterol excretion, and gene expression of major receptors, enzymes, and transporters involved in cholesterol metabolism, inhibiting the hepatic cholesterol synthesis [53, 64–72]

Reduction of hypertension	Antihypertensive effects: direct vasodilators, calcium channel blockers, inhibiting the gene expression of COX-2, controlling the vascular tone through the release of calcitonin gene-related peptide (CGRP), substance P (SP), and neurokinin A, increase in intracellular calcium, which causes the release of several neuropeptides [53–63]

Attenuating body weight	Increased energy expenditure, increased lipid oxidation, reduced appetite, reduced abdominal adipose tissue levels, decreased body fat, pad weights of epididymal and prerenal adipose tissues, and reduced fat accumulation by downregulating PPAR*γ* and C/EBP*α* in 3T3Li adipocytes [28, 49, 72]

Improving endothelial function	Improving endothelial function, inhibiting the transmembrane influx of calcium ions into cardiac and vascular smooth muscle, improving coronary vascular circulation, and decreasing expression of TRPV1 and cation influx [73–75]

Reduction in inflammation	Anti-inflammatory effects: lowering cytokines and C-reactive protein, reducing eNOS transcription, and depleting neurons of neurotransmitters, leading to reduction in pain sensation and blockade of inflammation [76–82]

^*∗*^Studies based on in vitro and in vivo studies and few human studies.

## List of Abbreviations

CAPs: Capsaicin/capsaicinoids
HSL: Hormone sensitive lipase
CPTI-*α*: Carnitine palmitoyl transferase-I*α*
UCP2: Uncoupling protein 2
CMSRF: Cardiometabolic syndrome risk factors
CHD: Coronary heart disease
CMS: Cardiometabolic syndrome
DHOC: Diabetes, hypertension, obesity, and coronary heart disease
Hsp27: Heat shock protein 27
NQO1: NAD[P]H: Quinone oxidoreductase 1
TRPV1: Transient receptor potential vanilloid receptor 1
VR1: Vanilloid receptor subtype 1
WHO: World Health Organization.
